# Anterior prostatic tumours are difficult to diagnose without MRI

**DOI:** 10.3332/ecancer.2012.252

**Published:** 2012-04-24

**Authors:** Giuseppe Petralia, Sarah Alessi, Ara Alconchel, Paul Summers, Gennaro Musi, Victor Matei, Ottavio De Cobelli, Giuseppe Renne, Massimo Bellomi

**Affiliations:** 1 Department of Radiology, European Institute of Oncology, via Ripamonti 435, Milan, Italy; 2 Department of Radiology, University Hospital Miguel Servet, Paseo Isabel la Catolica 1-3, Saragossa, Spain; 3 Department of Urology, European Institute of Oncology, via Ripamonti 435, Milan, Italy; 4 School of Medicine, University of Milan, via Festa del Perdono 19, Milan, Italy; 5 Department of Pathology, European Institute of Oncology, via Ripamonti 435, Milan, Italy

## Abstract

It is often uncertain whether a repeat biopsy is necessary in patients with at least one previous negative prostate biopsy but persistent suspicion of prostate cancer. Here we present the use of multi-parametric magnetic resonance imaging (mp-MRI) to successfully detect and localize a prostate cancer and we suggest that MRI can be useful in optimising repeat biopsy procedures of the prostate in patients with clinically significant carcinoma.

Prostate cancer (PCa) was readily detectable on multi-parametric magnetic resonance imaging (mp-MRI) of this patient as a mass located in the anterior aspect of the gland with bulging of the anterior prostate capsule. On high-resolution T2-weighted axial images, PCa appeared as a lenticular low-signal intensity lesion of the anterior fibromuscular stroma infiltrating into the transitional zone (Figure 1). The high cellularity of PCa impeded water diffusion, giving rise to the appearance of a hyperintense mass on diffusion-weighted (DW) images (b = 1000 s/mm^2^) (Figure 2a) with low apparent diffusion coefficient (ADC) value (dark grey) on the corresponding ADC map (Figure 2b). On dynamic contrast enhanced (DCE) MR images after contrast administration (8 mL of Gadobutrol IV, followed by 20 mL of saline flush, at rate of 2 mL/s), PCa appeared as focal enhancing lesion visible on the subtracted images (Figure 3a); the signal intensity/time curve of the PCa showed fast initial enhancement followed by wash-out (Figure 3b), suggestive of a leaky capillary bed.

This 61-year-old patient showed increasing PSA values over the last few years, up to 14 ng/mL (PSA velocity = 2.4 ng/mL/year, PSA DT (doubling time) = 3.1 years). For this reason, he had undergone several biopsy procedures (one in 2010 and three in 2011) with inconsistent results, as only one out of the four biopsies was positive for prostatic intraepithelial neoplasia (PIN).

After mp-MRI clearly detected and localised PCa, the subsequent biopsy was positive for acinar adenocarcinoma (Gleason 4+4) in 24 of the 55 samples obtained, all of them in the anterior aspect of the gland (bilaterally, mainly in the apex, but also in the mid and cranial levels), as indicated by mp-MRI. The patient is now scheduled for surgery.

There is an increasing role for mp-MRI of the prostate (including high-resolution T2, DW and DCE images) for optimising repeat biopsy procedures in patients with a clinically significant PCa [1].

## Figures and Tables

**Figure 1:**
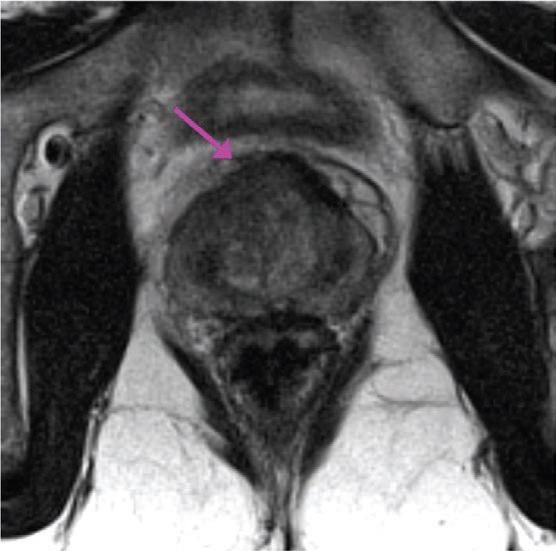
High-resolution T2-weighted image of the prostate. PCa appeared as a lenticular low-signal intensity lesion of the anterior fibromuscular stroma infiltrating into the transitional zone.

**Figure 2:**
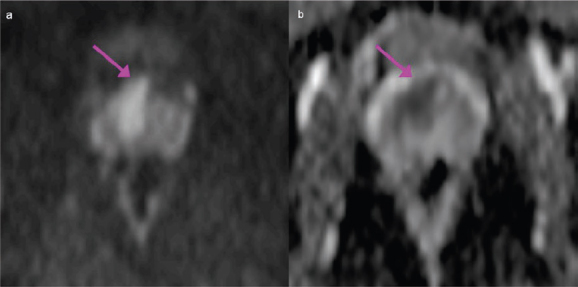
Diffusion-weighted (DW) image of the prostate. PCa appeared as a hyperintense mass on DW images (b = 1000 s/mm^2^), with low values (dark grey) on the corresponding apparent diffusion coefficient (ADC) map (mean ADC value ± standard deviation = 885.4 mm^2^/s ±159.5 mm^2^/s) (b).

**Figure 3:**
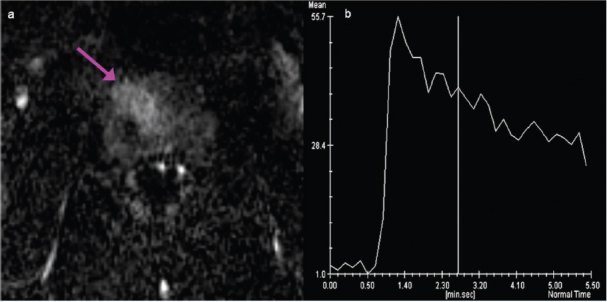
Subtracted dynamic contrast enhanced image of the prostate. PCa appeared as focal enhancing lesion (seen here in the 8th – 1st dynamic scan, around 50s after initiation of contrast injection) (a), with fast initial enhancement followed by wash-out (b).
